# Vaginismus: Sociodemographic profile and cultural aspects

**DOI:** 10.1192/j.eurpsy.2021.859

**Published:** 2021-08-13

**Authors:** S. Bader, W. Abbes, W. Mahdhaoui, W. Ltaif, L. Ghanmi

**Affiliations:** 1 Psychiatry, regional hospital of Gabed, gabes, Tunisia; 2 The Department Of Psychiatry, Hospital of gabes, Gabes, Tunisia; 3 Gynecology And Obstetrics, regional hospital of Gabed, gabes, Tunisia

## Abstract

**Introduction:**

Vaginismus is the most common reason for unconsummated marriages in Tunisia.

**Objectives:**

To describe the socio-demographic profile and to explore the clinical and cultural aspects of sexual functioning of women with vaginismus.

**Methods:**

It was a cross-sectional study established over a period of 3 months from the November 1st, 2019 to January 31st, 2020. This study focused on a population of women with vaginismus recruited from outpatient consultations of the hospital’s gynecology and psychiatry departments at the regional hospital of Gabes. We used a pre-established sheet exploring socio-demographic data, medical and gyneco-obstetric history and informations concerning the partner, the marital relationship and the woman’s sexual activity.

**Results:**

35 women were included. They had a mean age of 30 years, jobless (54.5%) and with a secondary or university education (91.1%). The mean duration of marriage was 2.4 years. Partner had mean age of 36 and suffering from sexual dysfunction (21.3%). Among women, 12.5% had been sexually abused, 51.6% had suffered “Tasfih”, 70% had attended discussions about painful defloration. Vaginismus was primary in 85.7% and total in 50% of the cases. About the received thoughts of the women, 40% thought that vaginismus requires medical treatment, 13 of them (40%) thought that the disorder could be resolved spontaneously and 20% believed in a story of witchcraft. 85% consulted a physician and 24.2% a traditional therapist.

**Conclusions:**

Vaginismus seems to be influenced by psychological and sociocultural factors so that a good psychoeducation of brides could reduce the incidence of this sexual disorder.

**Conflict of interest:**

No significant relationships.

